# Coupling EPR spin trapping of hydroxyethyl radicals and Strecker aldehydes analysis for predicting oxidative susceptibility of Chardonnay wines

**DOI:** 10.1016/j.crfs.2026.101413

**Published:** 2026-04-20

**Authors:** Pei Han, Alexandre Pons

**Affiliations:** a Univ. Bordeaux, Bordeaux INP, INRAE, OENO, UMR 1366, ISVV, Villenave d’Ornon, F-33140, France; b Bordeaux Sciences Agro, Bordeaux INP, INRAE, OENO, UMR 1366, ISVV, Gradignan, F-33170, France; c Seguin Moreau France, Z.I. Merpins, BP 94, Cognac, 16103, France

**Keywords:** EPR, 1-HER, Fenton, Strecker aldehydes, Wine aging, Oxidative susceptibility

## Abstract

During white wine oxidation, the Fenton reaction involves ethanol oxidation to form 1-hydroxyethyl radicals (1-HER) and triggers a complex oxidative cascade that produces Strecker aldehydes (SA), particularly methional and phenylacetaldehyde. We quantified free and total SA by GC-MS in 50 Chardonnay wines with different oxidative status. 1-HER kinetics were monitored by spin trap/EPR and expressed by *index a* (related to kinetic curve area/width). On average, the free form accounted for 21 % (methional) and 51 % (phenylacetaldehyde) of their total forms, while bound fractions varied widely. In oxidized wines, total methional correlates with the accumulation of the free fraction, whereas *index a* correlates with free methional. These observations were confirmed under controlled accelerated aging (AAg) conditions. Initial bound methional content predicted increases in free methional, supporting its value as a marker of oxidative sensitivity. Furthermore, using two spin traps (POBN and DMPO) with distinct radical selectivity, we confirmed that both 1-HER and hydroxyl radicals are implicated in methional formation. Overall, these results provide an enological interpretation of an EPR signature of oxidative susceptibility and suggest that combining *index a* with free SO_2_ may predict the oxidative shelf life of Chardonnay wine.

## Highlights

• Link between bound forms of Strecker aldehydes and quantitation of free forms during bottle aging.
• 1-HER and .•OH contribute to the formation of methional
• Relationship between EPR signature and methional concentration in Chardonnay wines.
• *Index a*, a potential oxidative stability indicator of methional formation.

## Graphical abstract

**Figure:**
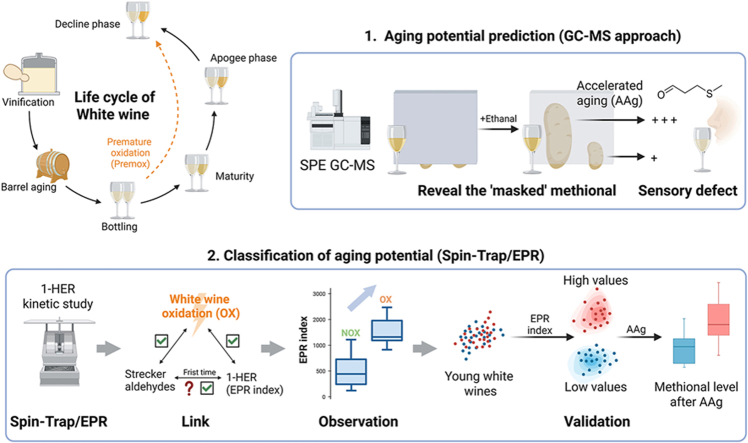

## 1 Introduction

Although widely consumed worldwide, high quality white wines are challenging to produce because of their sensitivity to oxygen and instability. Indeed, they may undergo premature aging, leading to the rapid loss of their varietal fruity notes and to the formation of off-flavors reminiscent of honey, bee's wax and cooked vegetables (Bueno et al., 2010; Escudero et al., 2000; Lavigne et al., 2008).

Much research in recent years has attempted to identify the main molecular markers and chemical mechanisms associated with their premature aging. Strecker aldehydes (methional and phenylacetaldehyde) play an important role in this process. Methional and phenylacetaldehyde generate notes that are reminiscent of boiled potatoes and old rose with a sensorial threshold of 0.5 μg/L (Escudero et al., 2000) and 30 μg/L (Sarrazin et al., 2007), respectively. In oxidized white wines obtained from long-term bottle aging, their level is quite well correlated, indicating similar formation mechanisms (Pons et al., 2021). On the other hand, the chemical mechanisms underpinning their formation are still debated. Yaylayan (2003) proposed a chemical mechanism involving the degradation of amino acids in the presence of dicarbonyl compounds, such as *ortho*-quinone, 2,3-butanedione (diacetyl) and 2,3-pentanedione. Oliveira et al. (2017) found that *ortho*-quinones contributed to the formation of phenylacetaldehyde from phenylalanine in synthetic wine conditions, while Nikolantonaki and Waterhouse (2012) showed that the reaction kinetics was very slow, suggesting the secondary importance of this pathway.

The formation of methional through quinone/methionine interactions in wine conditions is still not fully understood. Unlike phenylacetaldehyde, the formation of methional seems less due to phenolic/quinone species than to the contribution of reactive oxygen species (ROS) (Oliveira et al., 2017). In addition, Escudero et al. (2000) reported that these both aldehydes may also be formed through the oxidation of their corresponding higher alcohols such as methionol, which itself can originate from yeast or bacteria metabolism via the Ehrlich pathway (Hazelwood et al., 2008; Pripis-Nicolau et al., 2004).

Furthermore, methional and phenylacetaldehyde may be present in both free and bound forms owing to sulfite addition during vinification or prior to bottling (Bueno et al., 2014). The bound form is primarily linked to free bisulfite (free SO_2_) via a reversible sulfonate bond. The apparent equilibrium constants (K_app_) for methional (5.5 × 10^−4^ M) (Grant-Preece et al., 2013) and phenylacetaldehyde (8 × 10^−4^ M) (Grant-Preece et al., 2013) with bisulfite are quite high, making them relatively easy to dissociate. Indeed, these values are higher than that of acetaldehyde (1.5 × 10^−6^ M) (Burroughs and Sparks, 1973) which is known to form a stable adduct with bisulfite. Strecker aldehydes can also be released from the bound form in wine in the presence of other aldehydes, hydrogen peroxide (Grant-Preece et al., 2013), or more generally during oxidation, which induces a decrease in sulfite (Bueno et al., 2016).

Wine oxidation mechanisms are mediated by the production of free radicals, especially hydroxyl radical (•OH), which is a very reactive (i.e., short-lived) radical species (Rossetto et al., 2001, 2004). The technique known as spin trapping can be used to detect and quantify reactive radical species in wine by electron paramagnetic resonance (EPR) spectroscopy (Elias et al., 2009a). Using different spin traps, Elias et al. (2009a) demonstrated that the 1-hydroxyethyl radical (1-HER) was the main spin adduct detected, suggesting that the Fenton reaction (i.e., formation of the hydroxyl radical •OH and its subsequent oxidation of ethanol) is the major route for wine oxidation.

Nowadays, α-(4-Pyridyl *N*-oxide)-*N*-*tert*-butylnitrone (POBN) is widely used in white wines to detect carbon-centered radicals such as 1-HER (Elias et al., 2009a; Marchante et al., 2020a; Nikolantonaki et al., 2019a). When used simultaneously with Fenton reagents such as iron (II) and peroxide (H_2_O_2_), it enables the formation and quantification of radicals during oxidation. At room temperature, the formation kinetics of radicals follows a quasi-linear increase (Marchante et al., 2020b; Nikolantonaki et al., 2019a), whereas at 50 °C it fits a log normal model (Han and Pons, 2025). Radical formation/degradation kinetics can therefore be considered as a fingerprint of the oxidative stability of white wines.

The natural antioxidants found in grapes and in wines such as glutathione (GSH) (Pons et al., 2015a) or added by the winemaker such as ascorbic acid (Barril et al., 2016) and sulphur dioxide (SO_2_) (Danilewicz, 2021) are known to develop strong antioxidant properties, thereby improving the shelf life of the wine while delaying the loss of volatile thiols and the formation of volatile oxidation by-products (Pons et al., 2021). Recently, ellagitannins released by oak wood during wine maturation in oak barrels (Nikolantonaki et al., 2019b) as well as some peptides (Romanet et al., 2021) have been shown to contribute to the oxidative stability of wine, as they inhibit 1-HER. On the other hand, the pro-oxidant effect of (+)-catechin can induce an increase in the formation of free radicals (Elias et al., 2009b) and the formation of Strecker aldehydes (Pons et al., 2015a;Pons et al., 2015b).

Based on these observations, the antioxidant capacity of a white wine involves compounds or mechanisms that can slow down the formation of oxidation-related volatiles such as Strecker aldehydes. This raises a broader question: is this high antioxidant capacity associated with the potential of wine to age? Regarding beers, the brewery industry answered this question long ago by using EPR spectroscopy with spin trapping to predict their oxidative stability (Uchida et al., 1996). Subsequent sensory studies confirmed the relevance of this approach by linking the formation of free radicals in beer to the perception of off-flavors and the formation of (*E*)-2-nonenal, formed via the oxidative degradation of unsaturated fatty acids (Marques et al., 2017;Maxminer, 2016).

Using the recent development on EPR optimization to evaluate the kinetics of 1-HER (Han and Pons, 2025), this study goes further by assessing the shelf life of Chardonnay wines. It combines for the first time the application of two well-known techniques: 1-HER quantification by spin trap EPR and quantification of Strecker aldehydes (free and bound form) by GC-MS. Since the impact of the oxidation level of the wine on the EPR signature remains somewhat elusive, the knowledge gained may help in interpreting the kinetics of radical formation during Fenton/spin trap/EPR experiments.

## 2 Materials and methods

### 2.1 Chemicals

All chemicals were of HPLC grade. Hydrogen peroxide (30 % vol.), iron (II) sulfate heptahydrate (≥99.0 %), tartaric acid (≥99.7 %), acetaldehyde (99.5 %), methional (≥97 %), phenylacetaldehyde (≥95 %), 2-octanol (≥99.7 %), and sodium hydroxide (≥98 %) were purchased from Sigma-Aldrich (St. Louis, USA). The spin trap *α*-(4-pyridyl-1-oxide)-*N*-*tert*-butylnitrone (POBN, 98 %) was obtained from TCI (Zwijndrecht, Belgium). 5,5-Dimethyl-1-pyrroline-*N*-oxide (DMPO, ≥98 %) was obtained from Cayman Chemical (Michigan, USA). Sodium sulfate (≥99 %), ethanol absolute (>99.8 %) dichloromethane (≥99.8 %), methanol (≥99.8 %) and hydrochloric acid (37 %) were purchased from Fisher Scientific (Illkirch, France), and pentane (>99.7 %) from VWR (Pennsylvania, USA). Potassium metabisulfite was obtained from Laffort (Bordeaux, France). Water was purified by the Milli-Q system (Millipore, Saint-Quentin-en-Yvelines, France).

### 2.2 Wine samples

In total, the BIVB (Bourgogne Wine Board) selected and provided 91 Chardonnay commercial wines divided into three subsets. Wines from subset I (n = 3) was used to develop the forced-oxidation protocol and examine the contribution of 1-HER to Strecker aldehyde formation (Table 1). Subset II (n = 50) comprised young and bottle-aged wines (1998-2022) that were visually selected by enologists of the BIVB on the basis of the brownish color of the wine to obtain different oxidative levels (from low to high, Table S1). All wines were analyzed between 2023 and 2025. Subset III (n = 38; vintages 1998-2022) comprised young and bottle-aged wines (Table S2), spanning a wide range of bottle evolution and distinct from the wines included in Subset II. Subset III was composed of young wines (2019-2022; n = 24) and old wines (1998-2009; n = 9) including also some wines with intermediate aging (2016-2017, n = 5). The overall selection was used to assess the contribution of bound Strecker aldehydes to their free form during accelerated aging (AAg), whereas the two first groups were selected to evaluate the effect of AAg on free-radical formation kinetics. All these bottles were sealed with screw caps, microagglomerate corks and natural corks.

**Table 1:** Composition of Chardonnay wines (subset I) for experiments on Strecker aldehydes and 1-HER formation.

Wines	Vintage	EtOH a	TA b	pH	Tar. c	Free SO_2_^d^	OD_420_^e^	Test f
W1	2022	13.2	3.5	3.6	2.9	9.9	0.14	TSA
W2	2020	12.9	3.0	3.5	3.2	24.5	0.14	TSA
W3	2024	13.4	3.5	3.5	3.0	<3	0.02	FOx

a Ethanol content (EtOH % vol.).

b Total acidity (H_2_SO_4_ g/L).

c Tartaric acid (g/L).

d Free bisulfite (mg/L).

e Optical density at 420 nm.

f TSA: quantification of total Strecker aldehydes; FOx: Forced Oxidation, a rapid, short-term oxidative stress test triggered by Fenton oxidation.

### 2.3 Enological parameters

The oenological composition of wines was analyzed by Foss WineScan 79000 FTIR (FOSS, Nanterre, France). Optical density at 420 nm (OD_420_) was recorded using a UV-Vis Spectrophotometer V-730 UV-Vis (JASCO, Lisses, France). Free SO_2_ levels were determined using a Digital Wine Analyzer (Sentia™, Australia) with disposable test strips. Finally, the concentration of dissolved oxygen (DO) was measured using a NOMASense P6000 associated sensors Pst3 (PreSens, Regensburg, Germany).

### 2.4 EPR spin trapping method

The preparation of samples and the initiation of Fenton reaction are described in our previous work (Han and Pons, 2025). Briefly, 15 μL of acetaldehyde solution (567.5 mM in ethanol) was added to 10 mL white wine and incubated at 4 °C for 12 h to bind and remove free SO_2_. The treated sample (10 mL) was transferred to a 20 mL transparent vial equipped with a PSt3 oxygen sensor. Dissolved oxygen (DO) was removed by sparging with high-purity nitrogen at 10 mL/min through a microcapillary. DO was monitored in real time using an oxygen analyzer (NomaSense P6000). Sparging continued until the DO concentration was less than 0.1 mg/L, which typically took about 3 min. DO was removed before each EPR analysis. POBN (5.8 mg) was dissolved in 1 mL pretreated wine, followed by sequential addition of 5 μL of FeSO_4_ solution (10 mM) and 5 μL of H_2_O_2_ solution (120 mM) to initiate Fenton oxidation.

A disposable capillary micropipette containing the reactional mixture was inserted into the EPR cavity and analyzed by a EPR EMXnano spectrometer (Bruker BioSpin, Rheinstetten, Germany). The temperature of the cavity was controlled by a constant air flow via a controller (Noxygen, Elzach, Germany) in the resonance cavity at 50 °C, without any contact with wine samples. In our experimental conditions, the kinetic curves of POBN-HER formation adducts during the reaction in white wines follows a log-normal fit. Details concerning the integration of the data kinetics were previously described by Han and Pons (2025). The data were fitted from log-normal to obtain *index a* which is proportional to the ratio of the area under the curve (AUC) to the width of the peak at half-height. Higher values indicate greater radical formation within a shorter timeframe, whereas lower values suggest either lower total radical adduct formation or rapid decline in 1-HER adduct levels over time.

### 2.5 Analysis of Strecker aldehydes

#### 2.5.1 Quantification of free forms by SPE GC-MS

The extraction of volatiles was as described by Thibon et al. (2015). Extraction was carried out on a Gilson GX-274 ASPEC solid-phase extraction system (Villiers-Le-Bel, France). A Chromabond HR-X cartridge with phase on polystyrene-divinylbenzene hydrophobic polymer (6 mL, 500 mg, Macherey-Nagel, France) was activated by methanol (7 mL, 10 mL/min), and then equilibrated twice with 2 mL water/ethanol (90/10, v/v; 5 mL/min). A 10 mL sample spiked with 10 μL internal standard (IS, 60 mg/L 2-octanol) was then loaded onto the SPE cartridge (3 mL/min) after rinsing with water (2 mL, 5 mL/min) and dried by air pushing (8 mL, 6 mL/min). Then the analytes were eluted consecutively by 3 mL pentane/dichloromethane (50/50, v/v, 2 mL/min), and 3 mL dichloromethane/methanol (95/5, v/v, 2 mL/min). Finally, they were dried by freezing and concentrated in a nitrogen stream to a final volume of 100 μL for analysis.

The analysis was carried out using a gas chromatograph coupled to a mass spectrometer (Trace GC Ultra - DSQ II, Thermo Fisher Scientific, USA), equipped with a programmable temperature vaporizing (PTV) injector and an autosampler. Separation was carried out by SGE BP20 capillary column (50 m length, 0.22 mm inner diameter, 0.25 μm film thickness; Trajan, Victoria, Australia). The initial temperature of column was 40 °C then raised first at 10 °C/min up to 100 °C, held for 2 min, and later at 5 °C/min up to 230 °C for 2 min. The extract (0.5 μL aliquot) was injected in splitless mode (t = 1 min) with helium (≥99.9999 %, Messer) as the carrier gas. Acquisition processed by selected ion monitoring mode (SIM), ions *m/z* 104 and *m/z* 120 to quantifier the methional and phenylacetaldehyde. And internal standard (IS) 55 *m/z* for 2-octanol. The height of peaks was normalized by that of the IS 2-octanol then interpolated by curve of calibration with standard elaborated in Chardonnay wine.

#### 2.5.2 Optimization for total Strecker aldehydes analysis

Using the first observation by Grant-Preece et al. (2013) and other complementary approaches (Bueno et al., 2014; Zhang et al., 2019a), we developed a two-step approach to validate and analyze the total Strecker aldehyde content in Chardonnay wines: the first in wine model solution and the second in commercial wines.

The equilibrium of aldehyde bonding and release with acetaldehyde was studied in a wine model solution. A synthetic wine solution (SW) was prepared with miliQ water, containing ethanol (12 % vol.) and tartaric acid (5 g/L), with pH adjusted by sodium hydroxide (NaOH, 5M) to pH 3.5. Methional and phenylacetaldehyde were added to reach a concentration of 80 μg/L. Bisulfites were spiked in SW by potassium metabisulfite at 0.47 mM (equivalent to 30 mg/L free SO_2_) and incubated for 24 h to form bound Strecker aldehydes. Solutions were analyzed by SPE GC-MS.

Observations were validated with two different wines, W1 and W2, with different sulfite levels where bound forms were released by the following experiment. Ten mL of SW were spiked with acetaldehyde at 1 mM, 2 mM, 5 mM, 10 mM, 15 mM, 30 mM and 40 mM. Spiked samples were kept in the dark at room temperature for 24 h and analyzed by SPE GC-MS. Optimal conditions to reveal the bound forms of methional and phenylacetaldehyde in Chardonnay wines were acetaldehyde (15 mM) incubated for 24 h at room temperature. All tests were carried out in triplicate.

### 2.6 Accelerated process to age white wine

#### 2.6.1 Mild conditions, accelerated aging (AAg)

Accelerated aging (AAg) in laboratory conditions was conducted as follows: wine sample (80 mL) was bubbled with air at a low flow rate (approximately <1 mL/min) for 10 min at 20 ± 2 °C. This process decreased free SO_2_ to below 10 mg/L and increased dissolved oxygen to 7.5 ± 0.2 mg/L. The oxygenated sample was then placed in a brown SPME vial (20 mL) without head space and sealed with a gasket metal cap. All samples were placed in a vacuum bag and kept at 35 °C for 28 days in the dark. Samples were prepared in triplicate.

#### 2.6.2 Harsh conditions, forced oxidation (FOx)

Forced oxidation conditions were applied to wine samples to induce rapid SA formation. Briefly, 50 μL of FeSO_4_ (10 mM) and H_2_O_2_ (120 mM) solutions were added sequentially to 10 mL of wine aliquots. After that, POBN and DMPO (5 mM) were added individually or in combination. The tests were carried out in brown vials, which were sealed with a gasket metal cap, vortexed, and incubated for 2, 6 and 12 h at room temperature. After incubation, Strecker aldehyde levels were analyzed by SPE-GC-MS. All tests were carried out in triplicate.

### 2.7 Statistical analysis and data processing

Replicate data were screened for normality and homoscedasticity to select appropriate parametric or non-parametric procedures, and a logarithmic transformation was applied if necessary. For two-group comparisons, we used the Student t-test, the Wilcoxon test (paired, non-parametric) or the Mann-Whitney test (independent, non-parametric). For multiple pairwise comparisons, overall differences were assessed by ANOVA (assumptions met) or the Kruskal-Wallis test (non-parametric). When overall tests were significant, Tukey's HSD (after ANOVA) or Dunn's test (after Kruskal-Wallis) was used for post hoc pairwise comparisons. Differences were considered statistically significant at p < 0.05. Statistical analysis was performed using XLSTAT software (version 2023.3.1). The EPR kinetic curves were fitted by Sigma plot software (CA, USA). For data representations in box plot, horizontal line is the median. Lower (upper) box limits correspond to the first (third) quartile and whiskers correspond to the non-outlier minima and maxima. Outliers are indicated by symbols.

## 3 Results and discussion

### 3.1 Distribution of free and bound forms of Strecker aldehydes in chardonnay wines

The techniques developed to estimate or quantify total or bound aldehydes in white wines are based on the reversible binding with sulfite (de Azevedo et al., 2007). Released bound aldehydes with *p*-benzoquinone, followed by derivatization with PFBHA and quantification by SPE GC-MS/MS (Zhang et al., 2019a). We used the same approach with a protocol that mimics the slow release of key Strecker aldehydes in Chardonnay wines during bottle aging. There were several reasons for using acetaldehyde to reverse hydroxy sulfonate forms: its high level in wine (comprising about 90 % of the total), its formation pathway from oxidation of ethanol by Fenton reaction (Elias and Waterhouse, 2010), its increase during bottle aging (Escudero et al., 2025) and its high affinity for bisulfite that shifts the aldehyde-bisulfite equilibrium toward the release of bound Strecker aldehydes (Liu and Pilone, 2000).

#### 3.1.1 Optimization of total Strecker aldehydes quantification

To assay the total methional and phenylacetaldehyde concentrations, our preliminary tests were conducted on a synthetic wine solution (SW, Fig. S1) spiked with known amounts of methional, phenylacetaldehyde and free SO_2_. Bound forms of aldehydes were released by increasing concentrations of acetaldehyde. The methional concentration increased with acetaldehyde concentrations ranging from 1 to 5 mM. However, beyond 15 mM, the levels of methional and phenylacetaldehyde remained stable and did not differ significantly from those of the control sample. Therefore, bound Strecker aldehydes were released completely above this threshold. Additional tests were conducted in wines.

**Fig. 1:**
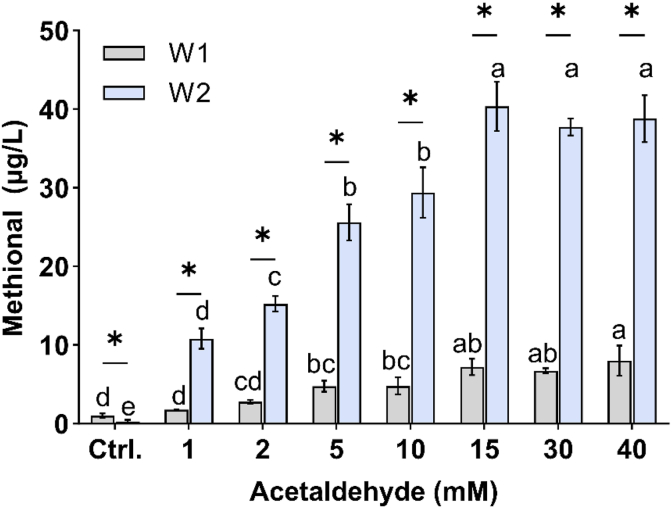
Impact of acetaldehyde addition on methional levels of two Chardonnay wines: W1 (2022, gray) and W2 (2020, blue). Control (Ctrl.): control wine without acetaldehyde addition. Lowercase letters and asterisk (∗) indicate differences between the four couples of wines (Student t-test, p < 0.05, n = 3) and levels according to the wine (Tukey test, p < 0.05, n = 3), respectively.

As shown in Fig. 1 and S2, increasing concentrations of acetaldehyde were added to two different wines (W1 and W2) containing different sulfite levels. After adding 15 to 40 mM of acetaldehyde, the methional and phenylacetaldehyde concentrations reached a maximum in both wines. Beyond this point, any additional increase in acetaldehyde concentration did not induce further changes in methional levels. In other words, there was no artefactual formation of SA due to the addition of acetaldehyde. We concluded that the optimal acetaldehyde concentration for assessing total Strecker aldehydes in white wine was 15 mM, a level which was then used for subsequent analysis. The results depicted in Fig. 1 also showed that even though W1 (control) had a significant higher free methional level than W2 (1.0 μg/L vs. 0.3 μg/L, p < 0.05), their total content was completely reversed, i.e. the total methional level in W2 was significantly higher than that in W1. Similar results were obtained with phenylacetaldehyde (Fig. S2).

#### 3.1.2 Distribution of Strecker aldehydes in chardonnay wines

**Fig. 2:**
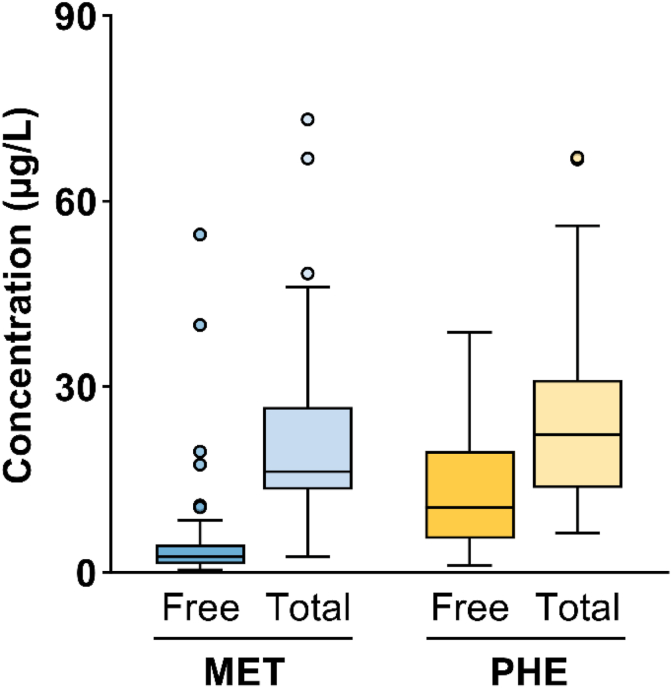
Box plot of free and total methional (MET) and phenylacetaldehyde (PHE) levels in Chardonnay wine with varying oxidation states (n = 50).

In total, 50 Chardonnay wines from various Burgundy appellations spanning the 1998 to 2022 vintages (Table S1), selected based on the color of the wine in the bottle (brownish hue), were analyzed to determine their free and total Strecker aldehyde content (Fig. 2). The concentration of free methional ranged from trace level 0.4 to 54.7 μg/L, while the total levels ranged from 2.5 to 73.2 μg/L. According to the wine, the bound form accounted for approximately 18 to 99 % of the total concentration. Free phenylacetaldehyde concentrations ranged from 1.2 to 38.8 μg/L, while the total level ranged from 6.4 to 67.1 μg/L. These concentrations are similar to those reported for different varieties and aging times, including Chardonnay wines, ranging from a few μg/L to 38.3 μg/L for methional and from 3.6 μg/L to 114 μg/L for phenylacetaldehyde (Bueno et al., 2014; Castejón-Musulén et al., 2022; Zhang et al., 2019b).

The bound form contributed from <1 to 94 % of the total concentration. This observation confirmed the presence of large amounts of the bound form of Strecker aldehydes in all Chardonnay wines, suggesting an impact on their release during wine aging as already suggested by Bueno et al. (2016) in wines.

#### 3.1.3 Distribution of Strecker aldehydes forms according to oxidation level: link with 1-HER formation kinetic

**Fig. 3:**
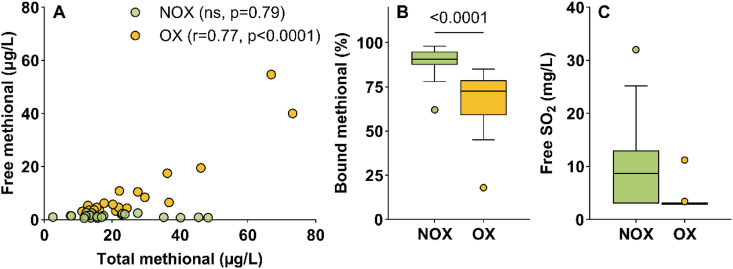
Distribution of different forms of methional in non-oxidized and oxidized Chardonnay wines, Subset II. (A) Correlation between total and free methional (Spearman, p < 0.05). (B) Percentage of bound methional and (C) free SO_2_ levels according to oxidation level. NOX: non-oxidized wines with free methional <3 μg/L (n = 26), OX: oxidized wines with free methional ≥3 μg/L (n = 24). Ns: not significant, p values indicate significant differences between groups (Mann-Whitney test, p < 0.05).

The wines from Subset II (Table S1) were classified into two groups according to their methional level. Thus, non-oxidized wines (NOX, n = 26) had low free methional levels (1.4 ± 0.6 μg/L), with all values < 3 μg/L, consistent with methional ranges reported for non-oxidized white wines, e.g., 1.08 ± 0.39 μg/L (Culleré et al., 2007) or trace to 3 μg/L in Chardonnay wines (Capone et al., 2018). These observations were consistent with the detection threshold of methional in model solution (2.4 μg/L) reported by Sarazin et al. (Sarrazin et al., 2007), even though a value of 0.5 μg/L is often found in the literature (Escudero et al., 2000). In contrast, oxidized wines (OX, n = 24) exhibited clearly higher concentrations, with values ≥ 3 μg/L (3.1 to 54.7 μg/L). We used 3 μg/L as an “analytical” oxidation threshold to differentiate the two groups. In the NOX group, bound and free aldehydes were uncorrelated (Fig. 3A) whereas total and free aldehydes were positively correlated (r = 0.77, p < 0.0001) as soon as oxidation occurred (OX group, n = 24). In OX wines, most of the free SO_2_ was below the detection limit of 3 mg/L (Fig. 3C) and the bound fraction dropped from 89.3 % (NOX) to 68.3 % (OX) (Fig. 3B).

Overall, the data suggest that during bottle aging, bound methional can serve as a reservoir, releasing methional from bisulfite adducts according to the oxidation level of the wine. A similar conclusion can be drawn for phenylacetaldehyde (Fig. S3). These results are consistent with those obtained in wines by Bueno et al. (2016) during forced aging.

**Fig. 4:**
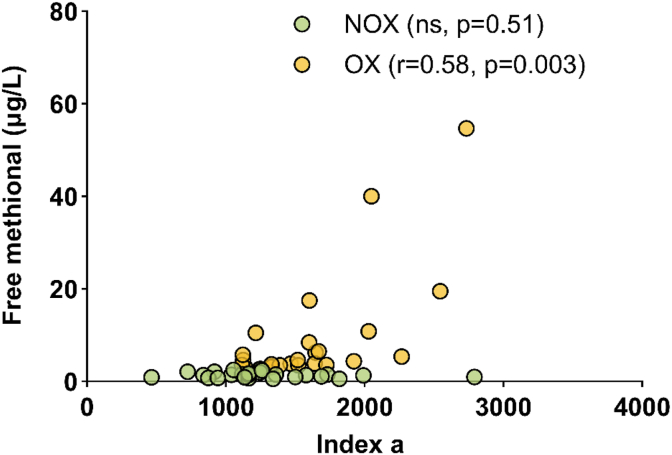
Spearman coefficients between *index a* and free methional according to oxidation level of young and old Chardonnay wines kept in bottle. (NOX: non-oxidized wines with free methional <3 μg/L (n = 26), OX: oxidized wines with free methional ≥3 μg/L (n = 24).

To go further, we recorded the formation kinetics of the 1-hydroxyethyl radical (1-HER) to calculate the *index a* of each curve and each wine (Fig. 4). Wines showed a wide range of *index a* value (dimensionless index). Free methional values and *index a* were not correlated in NOX wines, whereas a significant correlation emerged in the oxidized (OX) subset (r = 0.58, p = 0.003). These results suggest that the value of *index a* is linked with the oxidation mechanism occurring in white wine.

Both Strecker aldehydes were assayed in these experiments. However, we decided to focus on the link between *index a* and methional in the rest of this study, since the level of correlation with phenylacetaldehyde was very low (r = 0.26, ns) (Fig. S5). Usually, the evolution and distribution of both carbonyl compound concentrations during bottle aging are correlated (Pons et al., 2021). We confirm this trend within our data set (r = 0.74, p < 0.0001, Spearman test), having a great diversity in terms of origin, aging time and oxygen exposure. The lack of correlation between phenylacetaldehyde and *index a* illustrates the complexity of the oxidation process where subtle changes in the radical production may produce either methional or phenylacetaldehyde, depending on the composition of the wine, particularly its polyphenol composition (Oliveira et al., 2017).

### 3.2 Contribution of bound Strecker aldehydes to its free form during accelerated aging

**Fig. 5:**
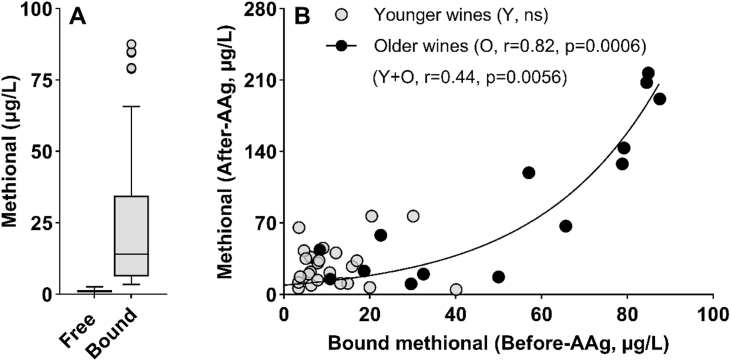
(A) Free and bound methional in control Chardonnay wines (Control) before accelerated aging (AAg). (B) Relationship between bound forms in control wine and free levels of methional after AAg (n = 38). Young wines from 2019 to 2022 (n = 24) and older wines from 1998 to 2017 (n = 14). Spearman correlations were calculated for younger (Y) and older samples (O). For old wines group, exponential growth fit is r^2^ = 0.87.

The accelerated aging protocol (AAg) was applied to 38 wines (Subset III) from different appellations, 24 young wines from 2019 to 2022 and 14 older wines from 1998 to 2017 (Table S2). Methional concentrations were quantified in all wines after the accelerated aging process (7.5 mg/L O_2_, 35 °C, 28 days). AAg conditions were inspired from previous works (Marrufo-Curtido et al., 2022). The wines selected for this experiment, from 1998 to 2017 vintages, contained low free Strecker aldehydes levels (mean free methional 1.3 ± 0.6 μg/L, free phenylacetaldehyde 5.6 ± 1.7 μg/L). On the contrary, we observed a wider variance for their bound form, 50.8 ± 29.8 μg/L (Fig. 5A) and 18.7 ± 10.3 μg/L (Fig. S4A) respectively for methional and phenylacetaldehyde.

After accelerated aging, the wines produced different levels of aldehydes according to the amount of bound form in the control wine (Fig. 5B). Of note, this variation did not appear to be related to free SO_2_ content, as no correlation (r = −0.28, p = 0.09) could be established between the content in the oxidized wines and its initial free SO_2_ levels before air bubbling. Furthermore, the increase in these aldehyde levels followed an exponential model (r^2^ = 0.87). This suggested a neoformation occurring during accelerated aging.

Regarding the age of the wines, we also observed that, as expected, the oldest were the sensitive to this treatment. Indeed, based on our selection, no significant correlation was obtained with the youngest, likely because, beyond the number of bound forms, these wines displayed a heterogeneous intrinsic antioxidant capacity, thereby protecting them from the release or neoformation of methional. Although a similar trend was observed for phenylacetaldehyde, its overall correlation was lower (r = 0.41, p = 0.0097) (Fig. S4). These results suggest that high levels of bound methional ‘hidden’ in wines could potentially predict its trend after oxidation.

### 3.3 Contribution of 1-HER to methional formation

To elucidate the relationship between the Fenton reaction and Strecker aldehydes, particularly the role of 1-HER (evaluated by the *index a*) in this process, the following experiments were conducted. First, a low free SO_2_ (<3 mg/L) young Chardonnay wine sample (vintage 2024, subset I) was rapidly oxidized by the Fenton reaction (H_2_O_2_/Fe^2+^) to assess its capacity for methional production. In spin trap/EPR experiments, POBN and DMPO are known to trap free radicals, particularly 1-HER (Elias et al., 2009a) and •OH (Márquez et al., 2019), respectively. In this experiment, we used POBN and DMPO as an exogenous ‘antiradical’ to observe the contribution of the Fenton reaction to the formation of methional.

Results showed that the concentration of methional in the wine in which the Fenton reactants were spiked increased significantly and rapidly after 2 h compared to control conditions. The levels remained high and quite stable during the experiment (12 h). On the contrary, the wine spiked with POBN (5 mM) or DMPO (5 mM) contained significantly lower levels of methional (Table 2). A mixture of the two spin traps prevented the oxidation of the wine and the formation of methional during the experiment, demonstrating the role of both hydroxyl and hydroxyethyl radicals in the formation of this aldehyde. The formation of phenylacetaldehyde by GC-MS was not monitored due to co-elution issues, when POBN was spiked to the wine.

**Table 2:** Impact of adding spin trap to a white wine on the formation of methional during Fenton oxidation.

Spin trap	Oxidation	Time				F	*p value*
		0 h	2 h	6 h	12 h		
Control	No	1.7 ± 0.5	-	-	1.7 ± 0.7		*ns*

POBN	No	2.3 ± 0.7	2.4 ± 0.7	1.9 ± 0.5	2.4 ± 0.6	0.42	*ns*
DMPO	No	1.3 ± 0.2	2.0 ± 0.5	2.0 ± 0.5	1.4 ± 0.1	4.06	*ns*
POBN + DMPO	No	2.6 ± 0.9	2.0 ± 0.9	1.6 ± 0.4	2.7 ± 0.8	1.29	*ns*

*P value*	No	*ns*	*ns*	*ns*	*ns*		

Control	Fenton	-	4.5 ± 0.5 a	5.3 ± 0.5 a	7.3 ± 0.5 a	43.78	*<0.001*
POBN	Fenton	-	2.3 ± 0.7 b	1.7 ± 0.3 c	4.9 ± 1.2 ab	7.39	*<0.05*
DMPO	Fenton	-	2.5 ± 0.4 b	3.6 ± 0.3 b	3.1 ± 0.6 b	31.59	*<0.001*
POBN + DMPO	Fenton	-	2.0 ± 0.2 b	1.9 ± 0.9 bc	3.1 ± 0.9 b	1.50	*ns*

*P value*	Fenton	*-*	*<0.01*	*<0.01*	*<0.01*		

Test carried out in wine W3 (2024). Fenton: wine spiked with Fenton reagents (Fe^2+^ and H_2_O_2_). Control: no spin trap addition. POBN or DMPO: wine spiked with POBN (5 mM) or DMPO (5 mM). POBN + DMPO: wine contained both spin traps (5 mM each). Lowercase letters indicate significant differences between treatments at the same time point (Tukey test, p < 0.05, n = 3).

**Fig. 6:**
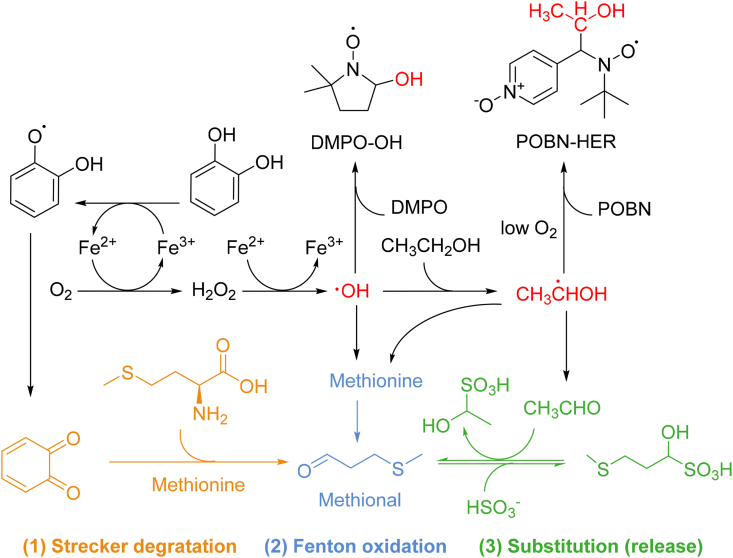
Schematic representation of methional formation pathway in dry white wines according to literature and our experiment with spin traps. (1) Neoformation of methional via Strecker degradation (Rizzi, 2006). (2) Hypothetical formation via direct Fenton type oxidation (Wietstock and Methner, 2013). (3) Release of methional from bound forms (Bueno et al., 2016; Grant-Preece et al., 2013).

A tentative explanation and schematic representation of these results is proposed in Fig. 6. The increase in methional in wine induced by the Fenton reaction could involve the Strecker degradation pathway of methionine and α-dicarbonyl compounds (quinone) (***Pathway 1***), as proposed in previous studies (Grant-Preece et al., 2013; Rizzi, 2006). However, under our experimental conditions considering the reaction rate, two additional pathways were primarily involved. In particular, the hydroxyl radical (Wietstock and Methner, 2013) or 1-HER generated by the Fenton reaction might directly oxidize methionine to produce methional (***Pathway 2***). Finally, the last pathway (***Pathway 3***) is based on the work of Bueno et al. (2016), and involves the formation of acetaldehyde produced by ethanol oxidation via the hydroxyl radical (Elias and Waterhouse, 2010). Such an increase in acetaldehyde could contribute to the release of bound methional from the wine. When both radicals are trapped, the reaction is stopped. This result sheds light on the link between the ability of wine to produce radical and methional and constitutes a first explanation of the link between radical production, the *index a* and free methional levels in old white wines (Fig. 4).

### 3.4 From 1-HER kinetic measurement of white wine to methional formation

Our findings raise a central question: are radical-formation kinetics related to a wine's susceptibility to oxidative change during bottle aging, which is in turn linked with oxidation-related volatiles?

**Fig. 7:**
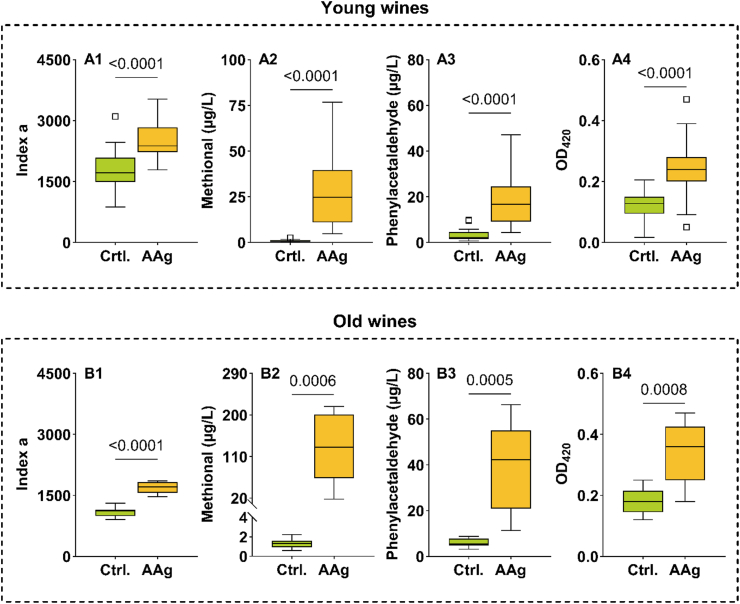
Box plot of distribution of values of *index a* (A1 and B1), free methional (A2 and B2), free phenylacetaldehyde (A3 and B3) and DO_420_ (A4 and B4) before (Ctrl.) and after accelerated aging (OX) in both young wines (A, 2019-2022, n = 24) and older wines (B, 1998 to 2009, n = 9). (Student T test, p < 0.05).

We analyzed several Chardonnay wines from Subset III: 24 young wines from 2019 to 2022 vintages and 9 oldest wines from 1998 to 2009 (Table S2) before and after accelerated oxidative aging (AAg, O_2_, 35 °C, 28 days). We determined the 1-HER evolution kinetic, absorbance at 420 nm (OD_420_), and the concentrations of free methional and phenylacetaldehyde (Fig. 7).

**Fig. 8:**
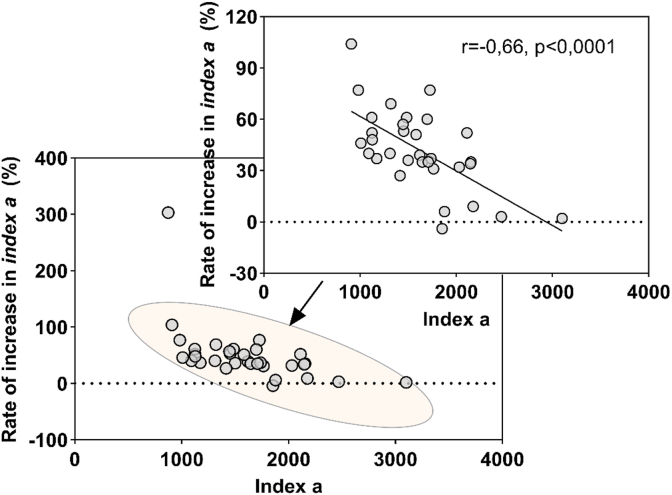
Link between *index a* in control wines and percentage increase after accelerated aging. The correlation was calculated for 32 wines (shown in the orange ellipse, Spearman test) after excluding an outlier. The solid black line represents the direction of correlation.

Most of the control wines exhibited *index a* < 2000, whereas average values were significantly higher after the treatment (2270 ± 528). In addition, Fig. 8 shows that the magnitude of change depends on the wine and its initial value, and that it follows an almost linear trend (r = - 0.66) for 32 wines after excluding an outlier. Wines with a low *index a* value seem to be much more sensitive to oxidative stress. On the contrary, wines with a high *index a* value are more stable. From an enological point of view, this means that, as expected, oxidized wines are less reactive to oxidation.

In parallel, we showed that Strecker aldehydes increased significantly (p < 0.0001): methional from 0.9 to 29.1 μg/L, phenylacetaldehyde from 3.2 to 17.3 μg/L and OD_420_ from 0.13 to 0.25. Similar trends were confirmed in the older wines. On average *index a* increased by 49.9 % after accelerated aging. Previously, we hypothesized *index a* to be an indicator of the oxidative susceptibility of white wines (Han and Pons, 2025). Our data validated this hypothesis while giving a first enological explanation of the new 1-HER dimensionless parameters useful to describe the oxidation of Chardonnay wines.

### 3.5 Predicting methional concentration based on *index a* value

Based on the data obtained in this work, we confirm the contribution of the bound methional form on the free odorant form in old Chardonnay wines aged in bottle for a long time. Accelerated aging (AAg) experiments further revealed the link between *index a* and the amount of free methional in the wine (Fig. 7). These results support previous observations and demonstrate, in the context of Chardonnay wine, that deciphering the methional pathway is quite complicated, as it involves several origins, including both chemical and biochemical pathways while this aldehyde also exists in an unstable, sulfite-bound form. In addition, Fig. 8 showed that an *index a* value of wines above 2000, corresponds to a slight increase after AAg (∼20 % on average). Using this threshold, we can also distinguish oxidized bottle aged wines, which exhibit significantly (p = 0.0065, Mann-Whitney test) higher methional concentrations in bottle (Fig. S6). To go further, we investigated the link between the 1-HER formation kinetics and its ability to produce methional after AAg using younger wines from subset III.

**Fig. 9:**
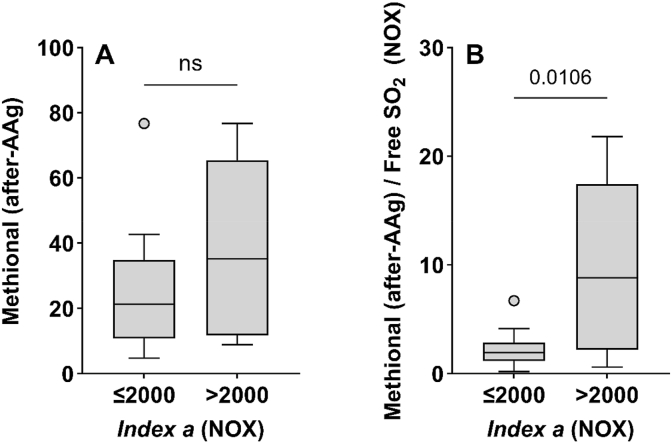
*Index a* threshold predicts susceptibility of young Chardonnay wines (vintages 2019-2022) to contain high methional levels after accelerated oxidation (AAg), *index a* ≤ 2000 (n = 17), *index a* > 2000 (n = 7). Results are presented (A) without considering free SO_2_ and (B) with free SO_2_ contribution (Mann-Whitney test, p < 0.05).

The main challenge was to integrate in our prediction model all these formation pathways while including the available reservoir of methional according to the concentrations in bisulfites. Given the diversity and complexity of the mechanisms involved, it is clear that no single measurement can fully address the question of aging potential associated with the amount of methional produced or released during aging. For this reason, based on spin trap/EPR analysis and *index a* determination, it was not possible to establish a threshold beyond which a wine can contain a high methional concentration after accelerated aging (Fig. 9A). Conversely, when the contribution of free SO_2_ in the wine is taken into account, we demonstrated that *index a* = 2000 represents a threshold above which the wine can generate higher methional concentrations when stored in the presence of oxygen (Fig. 9B). In our experimental conditions, we demonstrated that the combined determination of *index a* and free SO_2_ provides a relevant and practical assessment to measure oxidative stability.

Determination of shelf life of beverage is a tricky question that has been explored through different approaches. For example, spin trapping coupled with EPR and volatiles quantitation has been previously documented and tested in other beverages. For example, oxidative staling in beers involves multiple pathways, among which the Fenton reaction contributes to aldehyde formation (Andersen and Skibsted, 1998); linoleic acid oxidation leading to the formation of (*E*)-2-nonenal (Vanderhaegen et al., 2006). In this context, spin trap/EPR is particularly valuable, as it provides insights into the kinetics of radical formation and reflects the oxidative stability of beers through parameters such as lag time (Uchida et al., 1996). Accelerated storage modify the radical kinetic formation obtained by spin trap/EPR (expressed as area under curve, AUC, or lag time) as well as the formation of staling volatile markers, such as furfuryl ethyl ether (Valentoni et al., 2022) and (*E*)-2-nonenal (Maxminer, 2016). In addition, according to Marques et al. (2017), AUC is positively correlated with sensory staleness intensity of beers after accelerated aging whereas the link with known volatile molecular markers remains unclear.

More recently, Dekker et al. (2024), demonstrated the low but significant correlation between Oxygen Radical Absorbance Capacity (ORAC) of non-oxidized white wines and the formation of 2-aminoacetophenone (one of the oxidative related volatiles) during forced aging. It means that white wines having a low scavenging capacity expressed as low ORAC values, tend to produce much more 2-aminoacetophenone. In addition, Miao and Waterhouse (2025) proposed a method to estimate white wine shelf-life coupling free SO_2_, dissolved oxygen consumption, and the free SO_2_ depletion factor.

So, our results were in accordance with these last experiments and observations: based on our data set, we show for the first time that beyond an *index a* value of 2000, a Chardonnay wine with a low free sulfite content runs the risk of containing a high level of methional after accelerated aging. Therefore, such wines must be handled with great care, particularly with respect to oxygen exposure and free SO2 management during storage and aging.

## 4 Conclusion

In this work, we optimized a methodology based on the literature data to quantify free and bound forms of methional and phenylacetaldehyde. Fifty young and old Chardonnay wines were analyzed. We demonstrate the major contribution of methional in old oxidized wines, whereas the bound forms were widely distributed.

Accelerated aging in controlled conditions showed that the free methional concentration formed after oxidation was correlated with the amount of bound form. This relationship suggests that the amount of bound methional could serve as a potential predictor of the aging potential of wine. Additionally, we explored the relationship between the EPR signature (*index a*) and methional in wine. We found that oxidation induces its formation, at the same time, an increase in *index a*. Furthermore, the present investigation selected a spin trap approach, elucidating that radical oxidation (•OH and 1-HER) contributes to the formation of methional in white wine. In this regard, our results provide the first enological understanding of this dimensionless parameter, i.e. that it can serve as a proxy of the oxidative susceptibility of white wines. This finding was confirmed by a wide range of tests conducted both in the laboratory and on bottle-aged wines.

Together, these findings show how the Fenton/spin trap/EPR approach may be used to demonstrate that combining *index a* with the analysis of free SO_2_ levels could potentially predict the shelf life of chardonnay wines. These experiments were, of course, carried out in a laboratory under controlled conditions. Further investigations are needed to apply these results and knowledge to wines in real aging conditions.

## Author contributions

P.H.: conceptualization, methodology, investigation, writing, original draft, and writing review and editing.

A.P.: conceptualization, methodology, writing original draft, writing review and editing, supervision, and funding acquisition.

## Notes

The authors declare no conflict of interest.

## Declaration of competing interest

The authors declare no conflict of interest.
