# Supplementary material for: Coupling EPR spin trapping of hydroxyethyl radicals and Strecker aldehydes analysis for predicting oxidative susceptibility of Chardonnay wines
Source: Curr Res Food Sci. 2026 Apr 20;12:101413. doi: 10.1016/j.crfs.2026.101413 (PMC13126284; doi:10.1016/j.crfs.2026.101413)
Supplement: Multimedia component 1 [file mmc1.docx]

Coupling EPR spin trapping of hydroxyethyl radicals and Strecker aldehydes analysis for predicting oxidative susceptibility of Chardonnay wines

Pei HAN ^a, b^, Alexandre PONS ^a, b, c *^

AUTHOR ADDRESS

^a^ Univ. Bordeaux, Bordeaux INP, INRAE, OENO, UMR 1366, ISVV, F-33140 Villenave d’Ornon, France

^b^ Bordeaux Sciences Agro, Bordeaux INP, INRAE, OENO, UMR 1366, ISVV, F-33170 Gradignan, France

^c^ Seguin Moreau France, Z.I. Merpins, BP 94, 16103 Cognac, France

* Corresponding author

# APPENDIX

**Supplementary data**


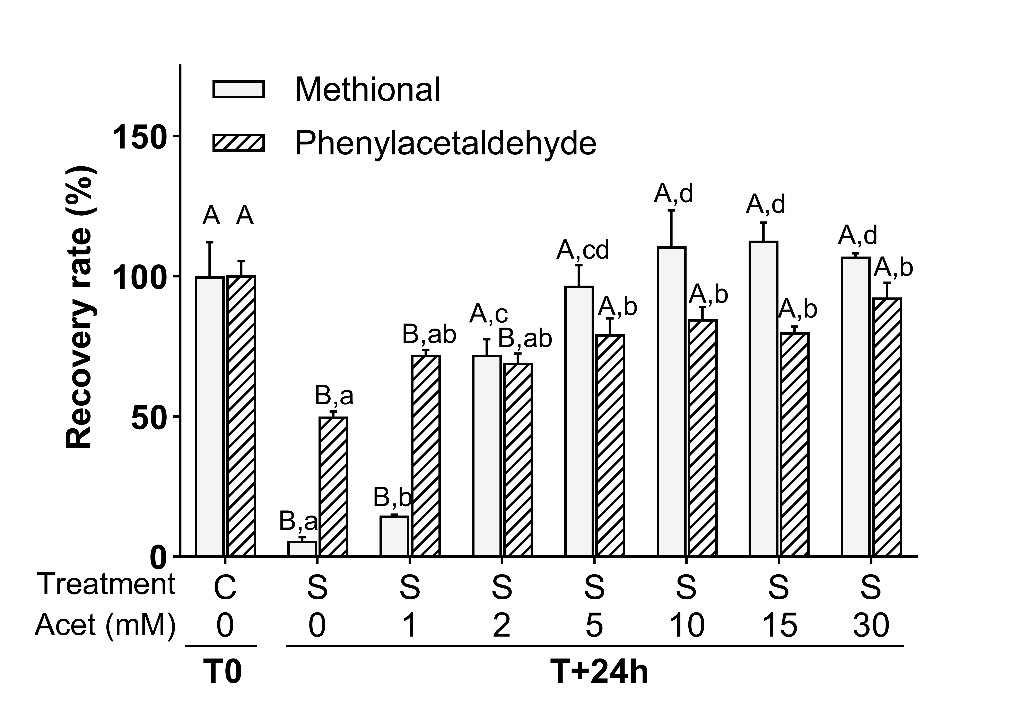


**Figure S1.** Release of Strecker aldehydes (methional and phenylacetaldehyde) following the addition of increasing concentrations of acetaldehyde (Acet) in a synthetic wine solution (SW). Control (C): SW containing aldehydes without incubation (T0). Treatment by bisulfite (S) addition (treatment with sodium bisulfite, 0.47 mM). Lowercase letters indicate significant differences between acetaldehyde addition levels after 24 h incubation. Capital letters indicate significant differences between T0 (control) and T+24 h for each treatment (n = 3, Tukey test, p < 0.05).


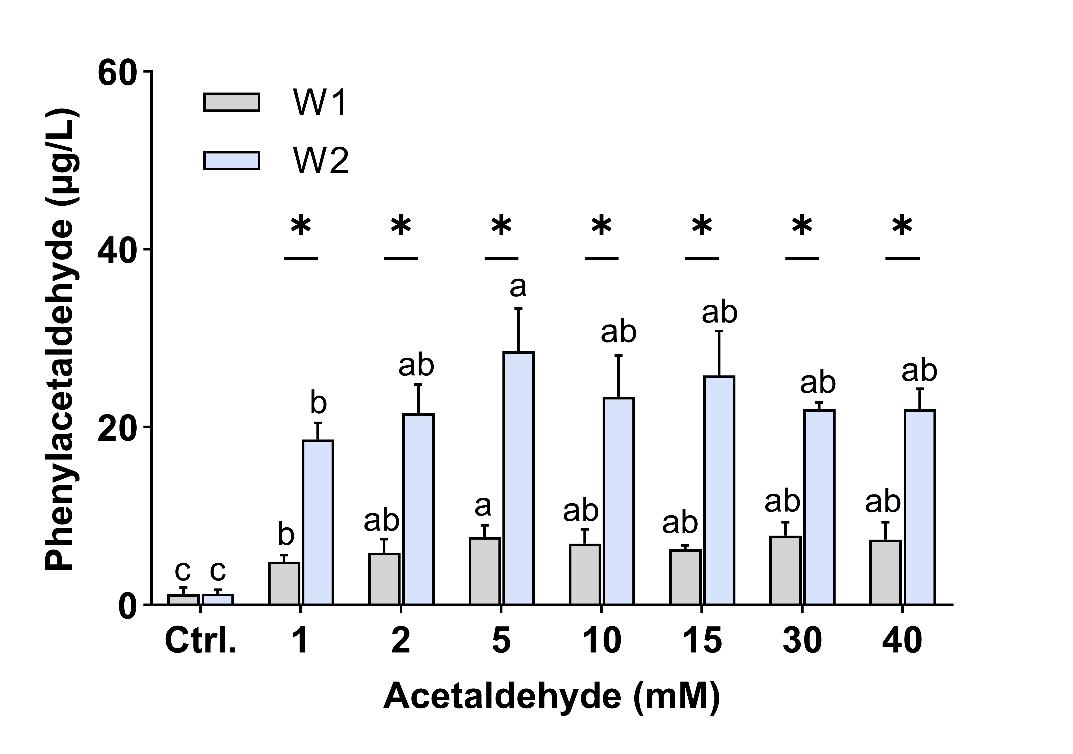


**Figure S2.** Assay of phenylacetaldehyde level by adding increasing concentrations of acetaldehyde (n = 3). Two wines were tested: W1 (2020, gray) and W2 (2022, blue). Control (Ctrl.): control wine without acetaldehyde addition. Lowercase letters indicate differences between acetaldehyde addition levels within each level (Tukey test, p < 0.05) and asterisk (*) indicates significant differences between the two tested wines at each acetaldehyde level (Student t-test, p < 0.05).

**Table S1.** Origin and composition of wines (subset II) for evaluating free and total methional and and their EPR signals.

| **ID** | **Vintage** | **Origin** | **Alc ^a^** | **AT ^b^** | **pH** | **ALac ^c^** | **Tar ^d^** | **DO_420_ ^e^** |
| --- | --- | --- | --- | --- | --- | --- | --- | --- |
| A1 | Côte de Beaune | 2022 | 13.0 | 3.1 | 3.6 | 1.2 | 2.7 | 0.20 |
| A2 | Côtes de Nuits | 2022 | 13.1 | 3.4 | 3.3 | 1.1 | 2.7 | 0.15 |
| A3 | Côte de Beaune | 2022 | 12.3 | 3.6 | 3.4 | 1.0 | 3.0 | 0.14 |
| A4 | Côte de Beaune | 2022 | 13.1 | 3.6 | 3.3 | 1.0 | 2.4 | 0.13 |
| A5 | Côte de Beaune | 2022 | 13.7 | 3.0 | 3.5 | 3.0 | 2.7 | 0.26 |
| A6 | Côte de Beaune | 2020 | 13.5 | 4.5 | 3.3 | Nd^+^ | 2.9 | 0.12 |
| A7 | Côte de Beaune | 2020 | 13.3 | 5.0 | 3.2 | Nd^+^ | 3.9 | 0.13 |
| A8 | Côte de Beaune | 2019 | 14.3 | 4.7 | 3.3 | 0.4 | 4.0 | 0.14 |
| A9 | Côte de Beaune | 2019 | 14.3 | 4.7 | 3.3 | 0.4 | 4.0 | 0.13 |
| A10 | Côte de Beaune | 2019 | 14.3 | 4.7 | 3.3 | 0.4 | 4.0 | 0.13 |
| A11 | Côte de Beaune | 2019 | 14.3 | 4.7 | 3.3 | 0.4 | 4.0 | 0.14 |
| A12 | Chablis | 2019 | 12.8 | 3.7 | 3.3 | 1.0 | 3.8 | 0.14 |
| A13 | Chablis | 2019 | 12.8 | 3.8 | 3.2 | 0.9 | 3.6 | 0.18 |
| A14 | Chablis | 2019 | 13.3 | 3.9 | 3.4 | 0.2 | 2.7 | 0.12 |
| A15 | Mâcon | 2019 | 13.6 | 3.6 | 3.4 | 1.0 | 3.4 | 0.11 |
| A16 | Chablis | 2018 | 12.9 | 3.5 | 3.5 | 1.8 | 2.8 | 0.21 |
| A17 | Chablis | 2018 | 12.4 | 3.2 | 3.3 | 0.9 | 3.0 | 0.09 |
| A18 | Côte de Beaune | 2017 | 13.9 | 4.0 | 3.4 | 1.2 | 3.6 | 0.03 |
| A19 | Côte de Beaune | 2017 | 13.9 | 4.0 | 3.4 | 1.0 | 3.5 | 0.10 |
| A20 | Mâcon | 2017 | 12.8 | 3.6 | 3.5 | 1.4 | 3.0 | 0.14 |
| A21 | Côte de Beaune | 2016 | 13.4 | 3.8 | 3.3 | 0.8 | 3.7 | 0.10 |
| A22 | Côte de Beaune | 2016 | 13.7 | 3.9 | 3.3 | 0.9 | 3.7 | 0.05 |
| A23 | Côte de Beaune | 2014 | 14.2 | 3.8 | 3.4 | 1.4 | 3.1 | 0.14 |
| A24 | Côte de Beaune | 2014 | 14.2 | 3.8 | 3.4 | 1.4 | 3.1 | 0.35 |
| A25 | Mâcon | 2014 | 12.9 | 3.7 | 3.5 | 1.7 | 2.7 | 0.20 |
| A26 | Champagne | 2013 | 11.9 | 5.8 | 2.9 | 3.1 | 5.6 | 0.17 |
| A27 | Champagne | 2013 | 11.9 | 6.1 | 2.9 | 3.1 | 4.6 | 0.16 |
| A28 | Champagne | 2013 | 11.9 | 6.1 | 2.9 | 3.1 | 4.6 | 0.16 |
| A29 | Mâcon | 2013 | 13.2 | 3.4 | 3.5 | 1.4 | 2.8 | 0.23 |
| A30 | Mâcon | 2012 | 12.9 | 3.5 | 3.5 | 1.3 | 2.4 | 0.22 |
| A31 | Côte de Beaune | 2010 | 13.5 | 4.1 | 3.2 | 1.7 | 2.5 | 0.18 |
| A32 | Côte de Beaune | 2010 | 13.5 | 4.1 | 3.2 | 1.6 | 2.5 | 0.22 |
| A33 | Chablis | 2010 | 13.4 | 4.6 | 3.3 | 3.0 | 3.1 | 0.24 |
| A34 | Côte de Beaune | 2010 | 13.4 | 4.6 | 3.3 | 3.0 | 3.1 | 0.24 |
| A35 | Côte de Beaune | 2010 | 13.5 | 4.6 | 3.2 | 1.4 | 3.4 | 0.40 |
| A36 | Beaune | 2008 | 12.9 | 4.2 | 3.4 | 2.8 | 2.6 | 0.23 |
| A37 | Chablis | 2005 | 13.5 | 3.7 | 3.4 | 1.7 | 2.9 | 0.21 |
| A38 | Chablis | 2005 | 13.5 | 3.7 | 3.4 | 1.7 | 2.9 | 0.29 |
| A39 | Côte de Beaune | 2005 | 13.9 | 3.8 | 3.3 | 1.0 | 2.5 | 0.31 |
| A40 | Côte de Beaune | 2005 | 13.9 | 3.8 | 3.3 | 1.0 | 2.4 | 0.33 |
| A41 | Côte de Beaune | 2005 | 13.5 | 4.2 | 3.3 | 1.6 | 3.0 | 0.33 |
| A42 | Côte de Beaune | 2005 | 13.6 | 4.2 | 3.3 | 1.5 | 2.9 | 0.42 |
| A43 | Côte de Beaune | 2002 | 13.6 | 4.3 | 3.2 | 1.5 | 3.0 | 0.21 |
| A44 | Côte de Beaune | 2002 | 13.6 | 4.3 | 3.2 | 1.4 | 2.9 | 0.34 |
| A45 | Côte de Beaune | 2000 | 14.0 | 4.0 | 3.3 | 1.5 | 2.9 | 0.26 |
| A46 | Côte de Beaune | 2000 | 14.1 | 4.0 | 3.3 | 1.5 | 2.9 | 0.27 |
| A47 | Côte de Beaune | 2000 | 14.1 | 3.9 | 3.4 | 1.7 | 2.7 | 0.28 |
| A48 | Côte de Beaune | 2000 | 14.0 | 3.9 | 3.4 | 1.7 | 2.7 | 0.32 |
| A49 | Côte de Beaune | 1998 | 12.9 | 3.7 | 3.3 | 1.6 | 2.1 | 0.13 |
| A50 | Côte de Beaune | 1998 | 12.9 | 3.8 | 3.3 | 1.6 | 2.1 | 0.12 |
| ^a^ Ethanol (% vol.). ^b^ Total acidity (H_2_SO_4_ g/L). ^c^ Lactic acid (g/L). ^d^ Tartaric acid (g/L). ^e^ Optical density (OD) at 420 nm. ^+^ Not detected. | | | | | | | | |


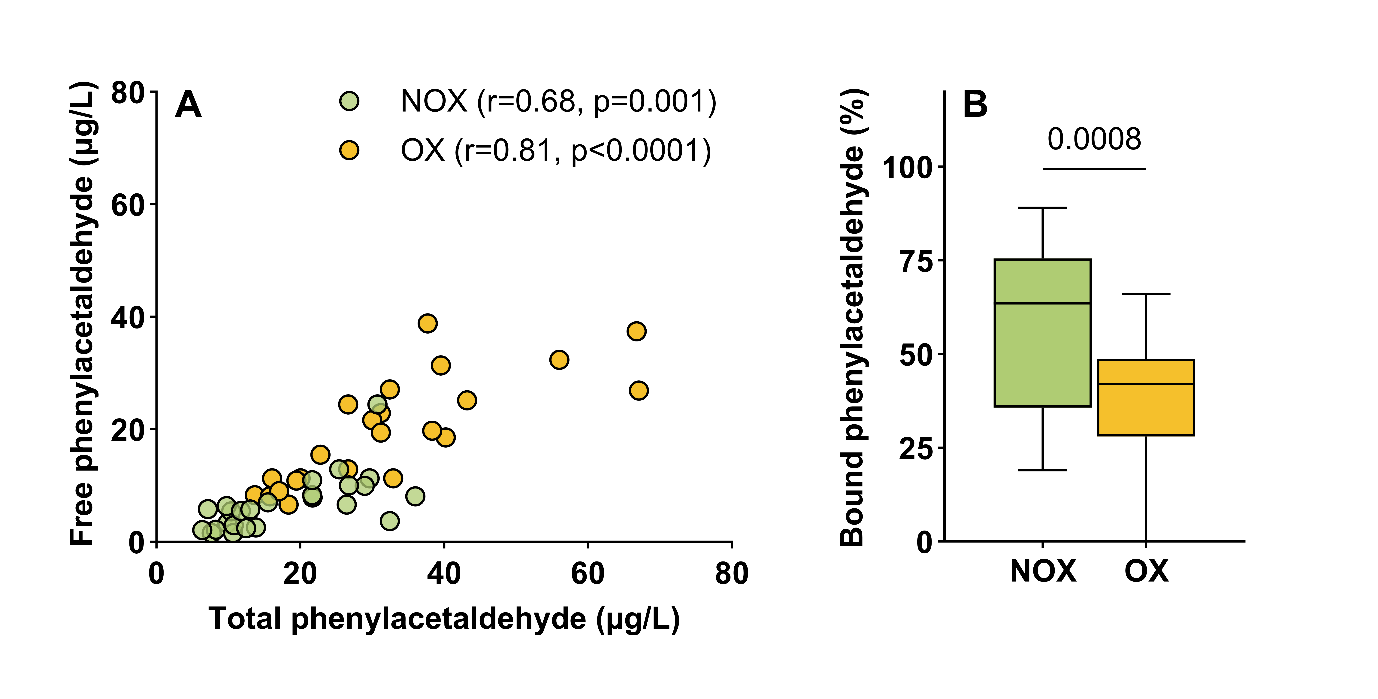


**Figure S3.** Distribution of different forms of phenylacetaldehyde in non-oxidized and oxidized Chardonnay wines (Subset II). (A) Correlation between total and free phenylacetaldehyde (Spearman, p < 0.05). (B) Percentage of bound phenylacetaldehyde according to oxidation level. NOX: non-oxidized wines (n = 26). OX: oxidized wines (n = 24): p value indicates significant difference between groups (Mann-Whitney test, p < 0.05).

**Table S2**. Origin and composition of wines (subset III) for evaluating of methional and impact of accelerated aging (AAg) and their EPR signals.

| ID | Vintage | Origin | Alc ^a^ | AT ^b^ | pH | ALac ^c^ | Tar ^d^ | DO_420_ ^e^ | SO_2_ ^f^ |
| --- | --- | --- | --- | --- | --- | --- | --- | --- | --- |
| S1 | 2022 | CN ^g^ | 12.8 | 5.1 | 3.1 | Nd. ^+^ | 3.7 | 0.09 | 13.0 |
| S2 | 2022 | CN | 12.9 | 5.0 | 3.1 | Nd. | 3.8 | 0.09 | 13.0 |
| S3 | 2022 | CB ^h^ | 13.3 | 4.3 | 3.3 | 0.1 | 3.7 | 0.13 | 5.4 |
| S4 | 2022 | CB | 12.6 | 3.8 | 3.3 | 0.6 | 3.7 | 0.14 | 10.3 |
| S5 | 2022 | Chablis | 12.9 | 3.7 | 3.4 | 1.4 | 3.7 | 0.20 | 3.0 |
| S6 | 2022 | CB | 13.2 | 4.3 | 3.3 | Nd. | 4.5 | 0.14 | 5.1 |
| S7 | 2022 | CB | 13.2 | 4.2 | 3.3 | 1.0 | 4.1 | 0.13 | 5.3 |
| S8 | 2022 | CB | 13.2 | 3.7 | 3.3 | 0.9 | 3.2 | 0.15 | 14.9 |
| S9 | 2022 | CB | 13.7 | 3.8 | 3.4 | 0.9 | 3.3 | 0.18 | 4.0 |
| S10 | 2022 | CB | 13.6 | 3.6 | 3.5 | 0.7 | 3.3 | 0.18 | 4.0 |
| S11 | 2022 | CN | 10.9 | 5.3 | 3.1 | 3.2 | 3.4 | 0.09 | 14.2 |
| S12 | 2020 | CC ^i^ | 13.0 | 4.7 | 3.2 | 0.7 | 4.4 | 0.17 | 5.6 |
| S13 | 2020 | CB | 13.0 | 3.9 | 3.3 | 0.7 | 3.4 | 0.13 | 24.5 |
| S14 | 2020 | Mâcon | 12.9 | 3.0 | 3.5 | 0.9 | 3.2 | 0.14 | 24.5 |
| S15 | 2020 | Mâcon | 12.7 | 3.9 | 3.3 | 0.7 | 3.1 | 0.12 | 17.1 |
| S16 | 2020 | CB | 13.1 | 3.7 | 3.4 | 0.6 | 4.0 | 0.11 | 12.4 |
| S17 | 2020 | BB | 12.6 | 4.4 | 3.1 | 1.1 | 4.1 | 0.08 | 5.5 |
| S18 | 2020 | CB | 13.4 | 4.1 | 3.3 | 0.4 | 4.3 | 0.07 | 8.3 |
| S19 | 2020 | Mâcon | 13.1 | 4.1 | 3.3 | 0.2 | 3.9 | 0.12 | 13.0 |
| S20 | 2019 | Chablis | 13.3 | 3.6 | 3.3 | 0.6 | 4.0 | 0.10 | 13.1 |
| S21 | 2019 | CB | 14.0 | 3.5 | 3.4 | 0.7 | 3.6 | 0.21 | 4.4 |
| S22 | 2019 | Mâcon | 13.7 | 4.5 | 3.3 | 0.2 | 3.6 | 0.02 | 17.7 |
| S23 | 2019 | Mâcon | 13.3 | 3.7 | 3.3 | 1.2 | 3.8 | 0.13 | 10.5 |
| S24 | 2019 | CB | 13.2 | 4.3 | 3.3 | 0.3 | 3.4 | 0.11 | 15.6 |
| S25 | 2017 | CB | 12.6 | 4.2 | 3.3 | 1.4 | 3.2 | 0.11 | 3.0 |
| S26 | 2017 | CB | 12.5 | 4.3 | 3.3 | 1.4 | 3.4 | 0.11 | 3.4 |
| S27 | 2017 | CB | 12.5 | 4.2 | 3.2 | 1.4 | 3.1 | 0.06 | 19.2 |
| S28 | 2016 | CB | 11.9 | 4.6 | 3.2 | 1.3 | 3.4 | 0.10 | 3.0 |
| S29 | 2016 | CB | 11.9 | 4.6 | 3.1 | 1.3 | 3.4 | 0.08 | 3.1 |
| S30 | 2009 | CC | 12.8 | 3.6 | 3.3 | 0.9 | 2.8 | 0.18 | 3.0 |
| S31 | 2007 | Mâcon | 13.2 | 3.6 | 3.4 | 1.7 | 2.6 | 0.21 | 5.0 |
| S32 | 1998 | CB | 12.9 | 3.7 | 3.3 | 1.7 | 2.1 | 0.15 | 3.6 |
| S33 | 1998 | CB | 12.9 | 3.7 | 3.3 | 1.7 | 2.1 | 0.15 | 3.6 |
| S34 | 1998 | CB | 12.9 | 3.7 | 3.3 | 1.7 | 2.1 | 0.14 | 6.7 |
| S35 | 1998 | CB | 12.9 | 3.8 | 3.3 | 1.6 | 2.1 | 0.12 | 13.5 |
| S36 | 1998 | CB | 14.1 | 4.0 | 3.3 | 1.6 | 2.9 | 0.22 | 8.6 |
| S37 | 1998 | CB | 14.1 | 4.0 | 3.3 | 1.5 | 2.9 | 0.20 | 7.8 |
| S38 | 1998 | CB | 14.1 | 4.0 | 3.3 | 1.5 | 2.9 | 0.25 | 9.5 |
| ^a^ Ethanol (% vol.). ^b^ Total acidity (H_2_SO_4_ g/L). ^c^ Lactic acid (g/L). ^d^ Tartaric acid (g/L). ^e^ Optical density (OD) at 420 nm. ^f^ Free bisulfites (mg/L). ^g^ Côte de Nuits. ^h^ Côte de Beaune. ^i^ Côte chalonnaise. ^+^ Not detected. | | | | | | | | | |
|  | | | | | | | | | |


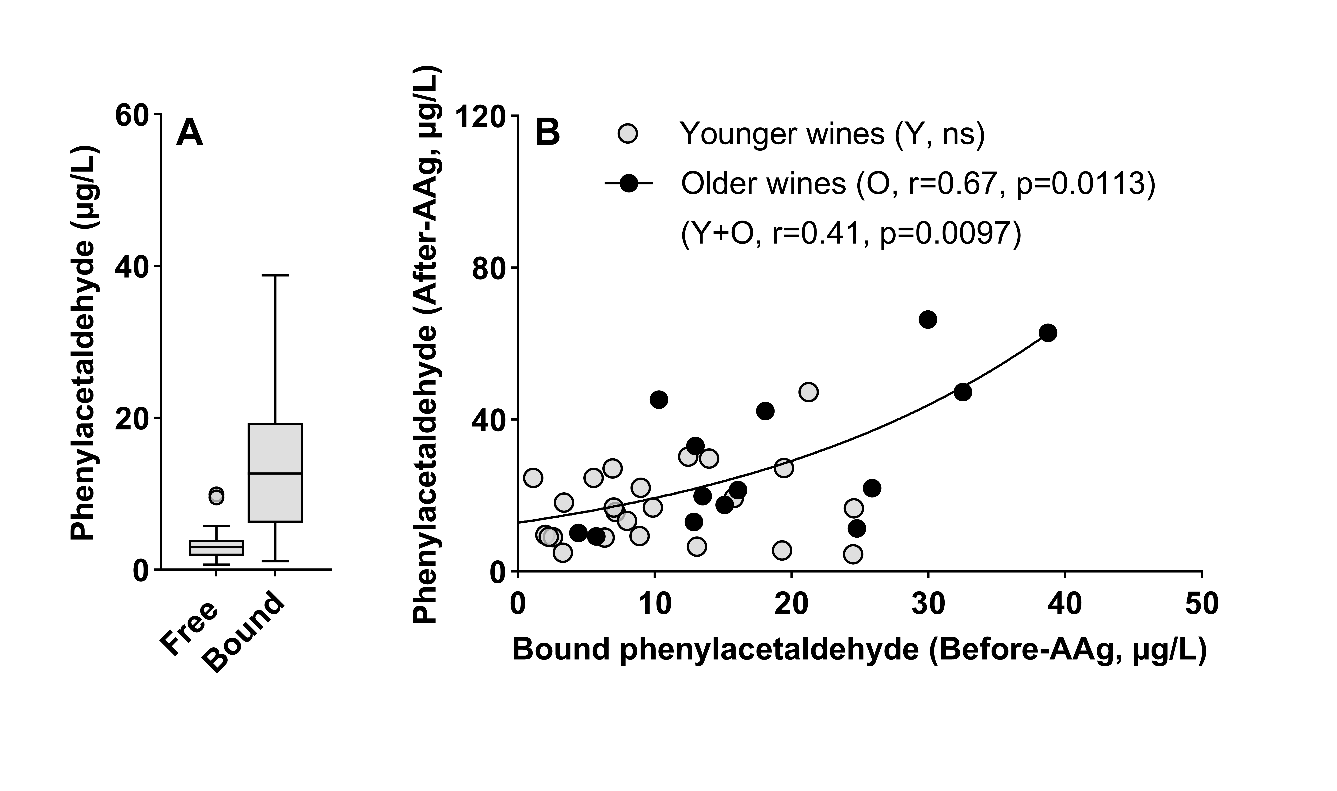


**Figure S4.** (A) Free and bound phenylacetaldehyde in control Chardonnay wines (Control) before accelerated aging (AAg). (B) Relationship between bound forms in control wine and free levels of methional after AAg (n = 38). Young wines from 2019-2022 (n = 24) and older wines from 1998-2017 (n = 14). Spearman correlations were calculated for younger and older samples. For older wines, exponential growth fits are r^2^ = 0.49.


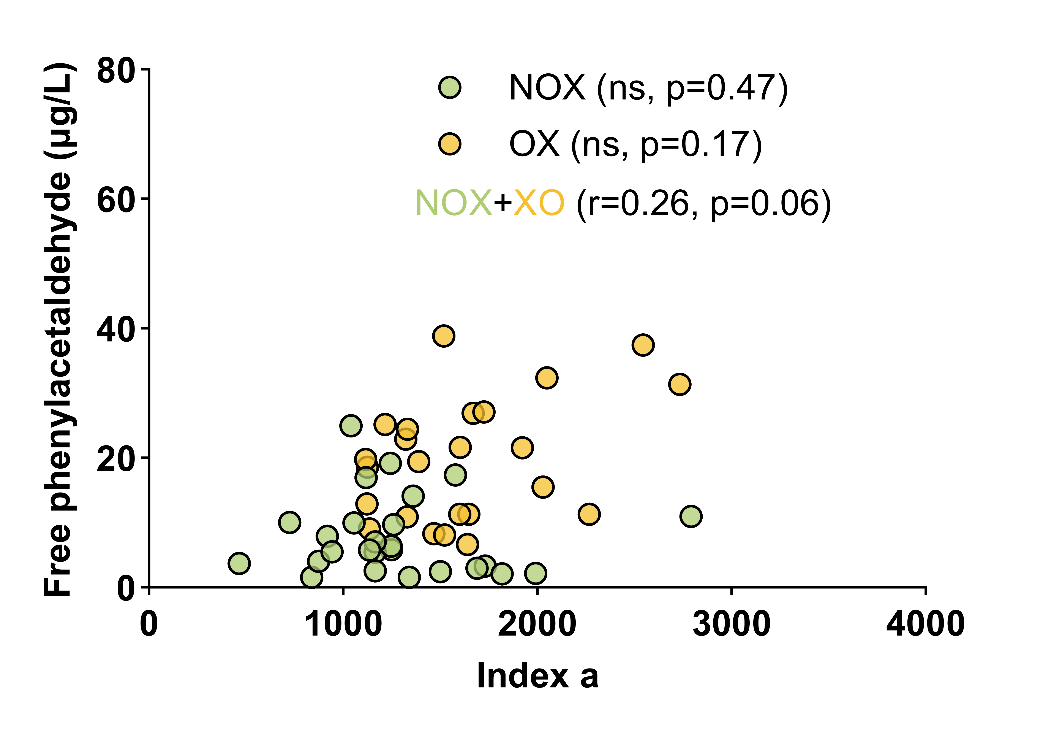


**Figure S5.** (A) Spearman coefficients between *index a* and free phenylacetaldehyde according to oxidation level. (NOX: non-oxidized wines (n = 26), OX: oxidized wines (n = 24). NOX+OX: correlations calculated using all wine samples from Subset II (n = 50). Ns: not significant (Spearman test, p < 0.05).


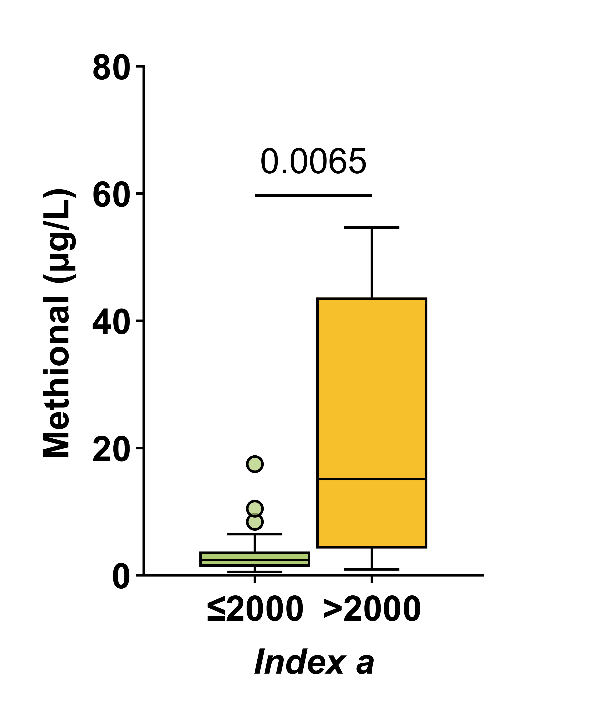


**Figure S6.** Boxplot of methional content according to *index a* values in young and old Chardonnay wines from Subset II (n = 50, Mann-Whitney test, p < 0.05).
